# Ferumoxytol MRA and non-contrast CT fusion in TAVR candidates with renal failure

**DOI:** 10.1186/1532-429X-18-S1-Q59

**Published:** 2016-01-27

**Authors:** Takegawa Yoshida, Fei Han, Ziwu Zhou, Olcay Aksoy, William M Suh, Peng Hu, J Paul Finn

**Affiliations:** 1 grid.19006.3e0000000096326718Department of Radiological Sciences, David Geffen School of Medicine at UCLA, Los Angeles, CA USA; 2 grid.19006.3e0000000096326718Division of Cardiology, David Geffen School of Medicine at UCLA, Los Angeles, CA USA

## Background

One of the advantages of CT angiography (CTA) over MRA for transcatheter aortic valve replacement (TAVR) planning is the ability to display aortic valve and arterial calcification accurately. However, in many patients who are TAVR candidates, renal impairment makes iodinated contrast media undesirable or contraindicated. Further, in patients with severe renal impairment, gadolinium based contrast agents may be problematic because of the perceived risk of NSF. We hypothesized that in patients where ferumoxytol-enhanced MRA (FEMRA) is a suitable alternative to CTA, vascular and valvular calcification may be accurately displayed over luminal anatomy by fusing non-contrast CT and FEMRA in these patients.

## Methods

Nine patients who underwent FEMRA for assessment of arterial access anatomy prior to TAVR had additional non-contrast CT of the thorax and abdomen. CT and MR DICOM data were processed in a commercially available software (Mimics V17.0, Materialize) and the calcium from the CT was isolated and registered to the FEMRA data.

## Results

**Figure 1:**
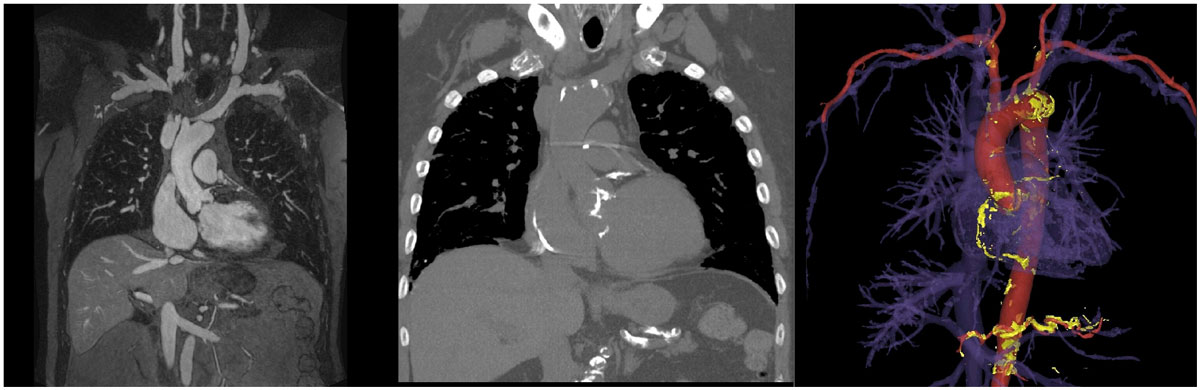
**FEMRA (left), non-contrast CT (middle) and Volume rendered FEMRA with calcium overlay (right) in an 84 year old male patient with calcific aortic stenosis who had successful TAVR placement**.

In all cases, the calcification was fused successfully with the 3D FEMRA data and volume rendered images were generated showing the degree and distribution of calcification clearly (figure 1), forming the basis for more confident procedure planning.

## Conclusions

By fusing non-contrast CT and FEMRA, both luminal anatomy and vascular calcification can be accurately defined, addressing one of the main limitations of MR over CT for TAVR planning.

